# Measuring Hair Cortisol Concentrations to Assess the Effect of Anthropogenic Impacts on Wild Chimpanzees (*Pan troglodytes*)

**DOI:** 10.1371/journal.pone.0151870

**Published:** 2016-04-06

**Authors:** Esther H. D. Carlitz, Robert Miller, Clemens Kirschbaum, Wei Gao, Daniel C. Hänni, Carel P. van Schaik

**Affiliations:** 1 Anthropological Institute and Museum, University of Zurich, Zurich, Switzerland; 2 Department of Psychology, Technische Universität Dresden, Dresden, Germany; 3 The Jane Goodall Institute Switzerland, Zurich, Switzerland; Centre for Cellular and Molecular Biology, INDIA

## Abstract

Non-human primates face major environmental changes due to increased human impacts all over the world. Although some species are able to survive in certain landscapes with anthropogenic impact, their long-term viability and fitness may be decreased due to chronic stress. Here we assessed long-term stress levels through cortisol analysis in chimpanzee hair obtained from sleeping nests in northwestern Uganda, in order to estimate welfare in the context of ecotourism, forest fragmentation with human-wildlife conflicts, and illegal logging with hunting activity (albeit not of primates), compared with a control without human contact or conflict. Concerning methodological issues, season [*F*(2,129) = 37.4, *p* < 0.0001, *r*^*2*^ = 0.18] and the age of nests [*F*(2,178) = 20.3, *p* < 0.0001, *r*^*2*^ = 0.11] significantly predicted hair cortisol concentrations (HCC). With regard to effects of anthropogenic impacts, our results neither showed elevation of HCC due to ecotourism, nor due to illegal logging compared to their control groups. We did, however, find significantly increased HCC in the fragment group compared to chimpanzees living in a nearby intact forest [*F*(1,88) = 5.0, *p* = 0.03, *r*^*2*^ = 0.20]. In conclusion, our results suggest that hair cortisol analysis is a powerful tool that can help understanding the impact of anthropogenic disturbances on chimpanzee well-being and could be applied to other great ape species.

## Introduction

Today, non-human primates face anthropogenic impacts of various kinds and the question how well and under which circumstances they can cope with human influence is of central importance for conservation programs. Consequently, conservation research aims at identifying the severity of anthropogenic impacts on primate behavior, health and physiology [1,2]. Concerning physiology, non-invasive stress monitoring through the endocrine stress marker cortisol is increasingly recognized as an important tool for the estimation of animal well-being and health on individual or population level [3]. In primates, the glucocorticoid hormone (GC) cortisol is secreted into the blood stream in response to various physiological or psychological stressors and plays a crucial role in enhancing catabolism in order to increase energy availability for the organism to cope with a stressor, while simultaneously decreasing anabolic pathways that are not essential for the immediate survival [4]. Consequently, increased cortisol secretion enables the organism to cope with short-term stressors. Unfortunately, elevated cortisol secretion over prolonged periods of time severely reduces individual fitness due to decreased immune response [5,6], the dysfunction of various organs [7,8], increased male and female infertility [9,10], and reduced growth [11].

To date, GC levels in feces [3,12–14] or urine [15] have been studied as non-invasive indicators of the severity of anthropogenic impacts on primates. However, for several reasons these methods require that animals are fully habituated to being followed by human observers, which severely limits their applicability: First, metabolite degradation within hours of defecation is an important confounder in these methods [14] requiring close proximity to the animals, and second, GC levels in urine and feces reflect only narrow time windows of hours or days, respectively, and therefore, measuring long-term stress levels in these matrices require repeated sampling of the same individual in order to even out short-term stress or biological rhythms. Yet, habituation of chimpanzees is a long process that can take up to seven years [16] and may have an impact on the animals itself [17,18]. Measuring long-term stress through cortisol concentrations in hair could overcome these problems. Like other great apes, chimpanzees build sleeping nests on a daily basis, which contain shed hair that can be gathered and analyzed for GCs, regardless of the animals’ habituation status.

Hair cortisol concentrations (HCC) are increasingly recognized as an integrated measure of the systemic cortisol secretion over several months. Many indirect validation studies [19–22] and three studies showing significantly increased HCC in consequence of multiple weekly ACTH injections [23–25] strongly suggest that HCC reflect the systemic cortisol secretion during hair formation (reviewed in [26–28]). Nonetheless, methodological constraints of HCC analysis arise from significant HCC differences across body regions [25,29–33] and waning cortisol concentrations towards the distal end of hair. Although the waning effect was mainly found in human hair [22,34–37], but not in animals [19,31], recent findings on chimpanzees from several zoos and one Ugandan sanctuary also confirmed the waning effect in this species, especially if animals were exposed to ambient weather conditions [29]. However, our investigations revealed that HCC waned along the hair shaft, while rank order was conserved across segments. Thus, we concluded that HCC measures from wild animals can still be used as long as the investigated length of hair is kept constant (e.g. the 3 cm close to the root). Concerning the body region effect, a confirmatory factor analysis verified that despite of differences in absolute values, HCC across the investigated body regions were driven by one common factor, presumably the systemic cortisol concentration, and HCC measures of all body regions provide similar biological information. We therefore concluded that it is possible to use shed hair from nests, which is a mixture of various body regions, albeit at the cost of a lower signal-to-noise-ratio. Thus, under the assumption that the shedding of hair is random in chimpanzees, an increased number of hairs found in the nest (e.g. 20 hairs per nest) will provide a stable mean of an individual’s average HCC [29]. Despite these methodological limitations, we found strong correlations between HCC and stress levels in these chimpanzees [38] as well as in European zoo orang-utans [19]. All these investigations suggest that HCC analysis can be a suitable, completely non-invasive tool to reflect the integrated stress level over a period of several months, because short-term stress and biological rhythms are leveled out.

Tourism is often considered a useful conservation tool because it protects the habitat at the same time as it promotes the local economy [39]. However, an increasing number of studies suggest that tourism can have adverse effects on the animals that shall be protected, e.g., reduced breeding success [40,41], decreased feeding times due to increased vigilance [42] increased risk of pathogen transmission [43], and generally increased stress levels [howler monkeys: [44,45], barbary macaques: [3], western lowland gorillas: [18], but see [46,47] for no influence]. Physiological data about the effect of tourism on chimpanzees is still missing although such information can contribute greatly to creating guidelines for truly non-invasive ecotourism [48].

While the majority of studies present mainly negative effects of tourism on animals, understanding other anthropogenic impacts on primates have produced varying results: Howler monkeys (hm) and spider monkeys (sm), for example, showed increased stress levels in the context of forest fragmentation (hm: [44,45,49], sm: [50]) and high human presence (hm: [51]) in some studies, whereas others found no influence of human presence and fragmentation on both species (hm: [50], sm: [51]). Similarly, research on different primate species in the same habitat elicited higher stress levels in disturbed habitats in gray-cheeked mangabeys [15] but not in two other ceropithecid species [52]. These ambiguous results illustrate that more research is needed in order to understand the impacts of anthropogenic disturbances on primate stress levels.

Direct observations suggest that chimpanzees show considerable behavioral flexibility and may be able to adjust to new environments and diets in human dominated landscapes [53–57], but only if locals are willing and capable of sustaining coexistence [58,59]. For example, Hockings and Sousa [60] suggested that traditional protection through folklore and religious practices towards chimpanzees can buffer adverse impacts of habitat destruction. However, physiological data that inform about the endocrine consequences of anthropogenic impacts in these contexts are still missing for chimpanzees.

The present study measured cortisol concentrations in shed hair from chimpanzee nests in order to examine whether ecotourism, illegal logging and forest fragmentation with severe human-wildlife conflicts resulted in increased long-term stress levels compared to chimpanzees without human impacts.

## Materials and Methods

### Study sites and sample collection

Permission for this research was granted by Ugandan National Council for Science and Technology (Ref.No.: NS 383) and the Uganda Wildlife Authority (Ref.No.: UWA/TDO/33/02). Samples were collected as naturally shed hair from chimpanzee sleeping nests in northwestern Uganda. These nests were found during transect walks for population counts (D.C. Hänni, unpublished data), through intensive search for nests, or in rare cases through following nest-building of habituated individuals. Collection took place during several dry seasons between 2012 and 2013 (Table 1) from four different communities with varying anthropogenic influence.

**Table 1:** Sample characteristics and descriptive statistics for hair cortisol concentrations of hair samples collected from chimpanzee sleeping nests in Uganda.

**Sample characteristics**							
Study groups	Control		Tourist		Fragment	Logging	
Season	Season 1	Season 2	Season 1	Season 2	Season 1	Season 1	Season 2
Forest	Budongo Forest Reserve (FR, intact)				Kasongoire (fragment)	Bugoma FR (intact)	
Sampling location (NE limit; SW limit)	N1°55.931’E31°44.035’; N1°55.604’E31°43.768’		N1°55.262’E31°43.276’; N1°53.906’E31°42.089’		N1°33.898’E31°32.880’; N1°33.564’E31°32.780’	N1°22.946’E31°04.745’; N1°14.283’E31°02.385’	
Collection period (estimated time represented by hair)	07–08.2013 (11–04.2013)	12.2013 (06–09.2013)	08.2012 (01–04.2013)	12.2013 (06–09.2013)	09.2012 (03–05.2013)	07.2012 (11–03.2012)	02–03.2012(07–10.2011)
Human impact	none (or minimal)		Tourist low season	Tourist high season	human-wildlife conflict		illegal logging + hunting (no primates)
Number of nests (new /recent/old)	49(7/21/21)	38(5/27/6)	29(14/11/4)	20(11/9/0)	14(14/0/0)	30(3/3/24)	15(2/6/7)
Nests assigned to individuals (m/f)			4 (3/1)	20 (17/3)	14 (7/7)		
**Descriptive statistics**							
Mean HCC (pg/mg)	0.52	1.63	1.07	1.18	2.35	0.3	0.8
SD	0.55	1.69	0.90	0.87	1.03	0.6	0.9
Minimum	0.04	0.08	0.11	0.19	0.90	0.04	0.04
Maximum	2.63	6.32	4.31	3.21	4.12	1.79	3.48

Hair samples were gathered from two communities in Budongo Forest Reserve: (a) one community without human impact (control group) and (b) the Kaniyo Pabidi chimpanzee community (tourist group) that is being visited by tourists more regularly since 2006. Chimpanzees were usually visited by tourists during two hours per day (one hour during morning and afternoon) with the number of visitors fluctuating considerably between days and seasons (own observations). If capacities were available, two tourist field guides would stay with the chimpanzees for up to five hours per day for further habituation. During the time of sample collection, the tourist community consisted of more than 90 identified individuals including babies (tourist field guide Joshua Ezua, pers. comm.). Hair samples for the control and tourist group were collected during two seasons, one season that reflects lower touristic activity in the hair (season 1: between November and April) and one that includes the two months peak tourist season (season 2: between June and September).

Further samples were collected from (c) a small community (27 individuals including eight babies and juveniles) next to Kasongoire village and approximately 15 km south of Budongo Forest Reserve. These chimpanzees live in small riverine forest fragments inside the sugar cane plantation of Kinyara Sugar Ltd. While fresh hair samples from known individuals were collected in September 2012 (represented time period in hair samples: March-May), the behavior of this group was observed between April and May 2012 during 175.5 h using 15 min scan sampling with special focus on human-wildlife interactions in order to identify whether the activity budged of Kasongoire chimpanzees differed from that of wild habituated groups living in intact forests (Budongo Conservation Field Station—BCFS unpublished report to World Wide Fund for Nature—WWF, May 2012). Additional nest samples were collected from chimpanzee nests in (d) Bugoma Forest Reserve where illegal logging and hunting (though not of primates) takes place (D.C. Hänni, unpublished data). Bugoma hair samples reflected similar periods of the year as the control and tourist groups (season 1: November-March; season 2: July-October, see Table 1 for details on hair collection periods).

From each nest we collected at least 20 single hairs (preferably more than 25 hairs) longer than 4 cm. Hair samples were picked with flamed tweezers and put into a dry envelope which was then kept in a sealed plastic container with silica gel until sample preparation. For each nest we recorded the GPS-coordinates, date of collection, nest height, tree species and nest age. For nest age estimates, nests were divided into three categories: new nests (leaves mostly green and flexible), recent nests (leaves green to brown and dry), old nests (leaves brown and partly decaying). Nest characteristics for each group are listed in Table 1.

### Hair cortisol analysis

From each hair, the proximal 0.3 cm of the hair shaft (including hair root for which genetic analyses is planned) as well as the distal part of hair segments longer than 3.3 cm were cut off and were excluded from hormone analysis. The remaining 3 cm long hair shafts were prepared for hormone analysis. If more than 60 hairs were available, hair shafts were segmented into four 1-cm-segments prior to analysis.

The procedures for washing and steroid extraction followed the protocol described by Gao and colleagues [61], who analyzed hair steroids with liquid chromatography tandem mass spectrometry (LC-MS/MS). One change was made to the protocol: The dry residue was resuspended using 175μL distilled water. Afterwards 100μL, not 150 μL, of the medium were injected into a Shimadzu HPLC system (Shimadzu, Canby, OR, USA) coupled to an AB Sciex API 5000 Turbo-ion-spray® triple quadrupole tandem mass spectrometer equipped with Atmospheric Pressure Chemical Ionization (APCI) Source (AB Sciex, Foster City, CA, USA). The system was controlled by AB Sciex Analyst® software (version 1.5.1). The lower limit of detection was ~0.1pg/mg. Intra- and inter-plate coefficients of variance ranged between 3.7–8.8%. All samples were prepared and analyzed within the same time period in order to prevent batch effects.

### Statistical analysis

HCC data were not normally distributed but approximated normal distribution with Box-Cox transformation. Thus, transformed data were used for inferential statistics (transformation coefficient *λ* = 1/7; [62]).

For methodological investigations, we explored whether the HCC waning effect was present in chimpanzee shed hair from representative 12 nests and whether nest age had an effect on HCC using a multilevel model for all *i* individuals (HCC^1/7^ = Segment + Individual_i_) and a one-way ANCOVA with chimpanzee group as a covariate (HCC^1/7^ = Nest Age + Chimpanzee Group), respectively. Throughout all subsequent analyses, nest age was used a covariate.

A two-way (chimpanzee group x season) ANCOVA was carried out to examine the effect of tourism and logging. A one-way ANCOVA was employed to examine whether forest fragmentation with human-wildlife conflicts increased HCC levels of Kasongoire chimpanzees compared to HCC levels of the nearby Budongo chimpanzees that were sampled during comparable time periods of the year (season 1 of control and tourist group). All analyses were performed using R 3.1.3 [63] statistical software.

## Results

Sample characteristics and descriptive statistics are presented in Table 1. For estimations on time periods represented by each hair sample, we subtracted an estimated 10 weeks shedding time after hair growth stops (on average 2–3 months in humans, [64]) and the estimated nest age from the date of sample collection. All relevant data for this paper are provided in S1 Table.

Of the 195 nest samples, 48 (19 recent, 29 old nests) were below the detection limit. However, the following test statistics obtained the same results with or without these samples (data not shown). Thus, we decided to include them because they still contained valuable information (apparently low concentrations).

**Fig 1:**
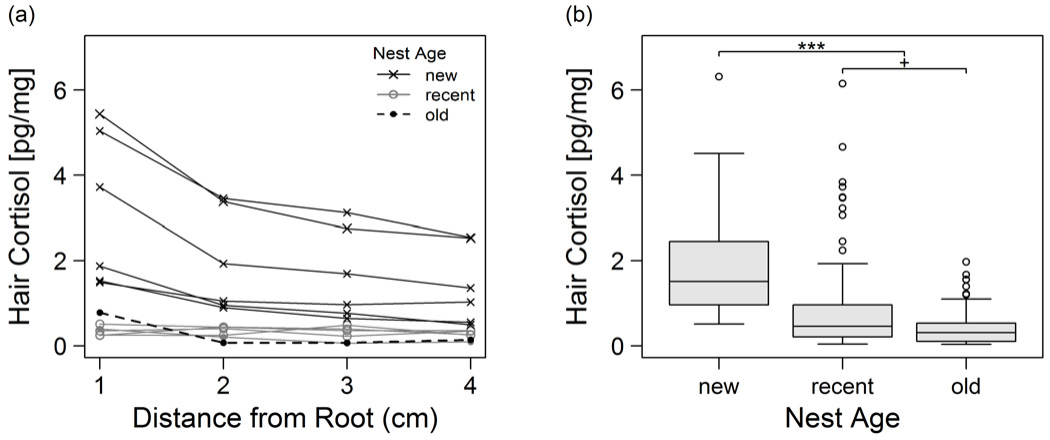
**Illustration of (a) waning and (b) nest age effect on hair cortisol concentration.** (a) Line plot illustrating hair cortisol concentrations (HCC) along four consecutive 1-cm-segments. Data are shown for a representative subsample from 12 chimpanzee sleeping nests with different nest age classes. HCC was significantly different between segments [*χ*^2^(3) = 27.7, *p* < 0.0001, *r*^2^ = 0.07] with HCC decreasing towards the distal end of the hair shaft. (b) Boxplots with 1.5 IQR showing HCC from 181 sleeping nests depending on the age class of the nest during hair sampling. Planned contrasts indicated that HCC was significantly higher in new vs. recent and old nests [*t*(2) = 6.3, *p* < 0.0001, *r*^2^ = 0.95] whereas HCC was only borderline significant between recent and old nests [*t*(2) = 1.85, *p* = 0.07, *r*^2^ = 0.63].

HCC was significantly different between segments [*χ*^2^(3) = 27.7, *p* < 0.0001, *r*^2^ = 0.07] with HCC decreasing towards the distal end of the hair shaft (Fig 1A). Consequently, only the proximal three cm of hair were used for analysis in order to control for this waning effect. Furthermore, HCC decreased with increasing nest age [*F*(2,178) = 20.3, *p* < 0.0001, *r*^2^ = 0.11, Fig 1B]. Planned contrasts indicated that HCC was significantly higher in new vs. recent or old nests [*t*(2) = 6.3, *p* < 0.0001, *r*^2^ = 0.95], whereas the difference in HCC was only borderline significant between recent and old nests [*t*(2) = 1.85, *p* = 0.07, *r*^2^ = 0.63]. Thus, nest age was included as a covariate in the following analyses.

**Fig 2:**
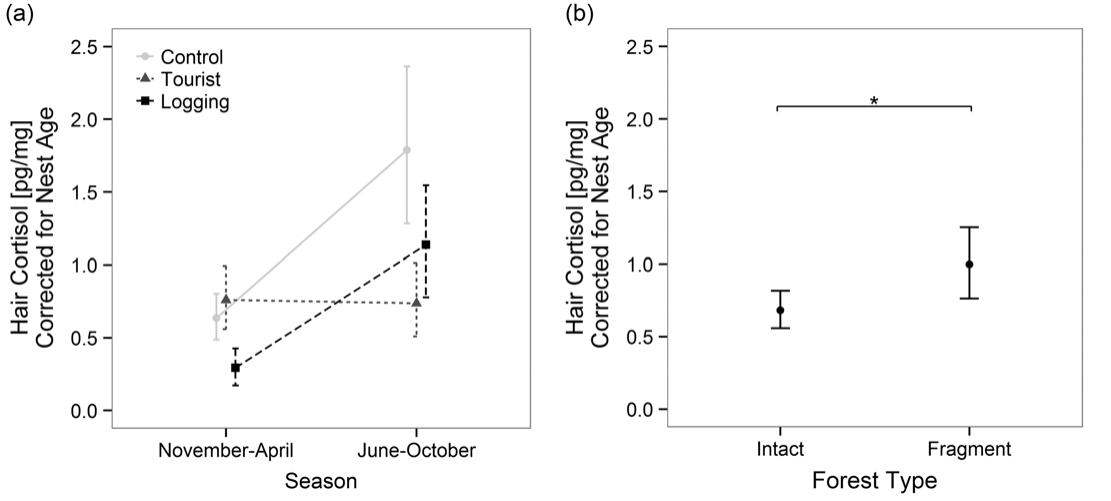
Differences in hair cortisol concentrations between seasons and chimpanzee communities with diverse anthropogenic impacts. Residualized mean hair cortisol concentrations (accounting for nest age effect) with 95% CI in different chimpanzee groups and seasons. (a) Chimpanzees did not exhibit significantly more cortisol due to tourism or logging in comparison to the control group without human contacts. A significant effect of seasonality [*F*(2,129) = 37.4, *p* < 0.0001, *r*^2^ = 0.18] was presumably unrelated to human impacts. (b) The comparison of hair cortisol concentration between chimpanzees living in an intact forest and those in a forest fragment with severe human-wildlife conflicts revealed significantly elevated HCC in the latter group [*F*(1,88) = 5.0, *p* = 0.03, *r*^2^ = 0.20].

The comparison of HCC between tourism and control chimpanzees revealed significantly higher HCC in season 2 (June-October) in the control chimpanzees [*F*(3,132) = 32.6, *p* < 0.0001, *r*^2^ = 0.16]. Furthermore, a significant effect with small effect size was found for chimpanzee group [*F*(3,132) = 4.7, *p* = 0.03, *r*^2^ = 0.05]. However, analyses also showed a significant group x season interaction effect [*F*(3,132) = 11.2, *p* < 0.01, *r*^2^ = 0.05] as a result of elevated HCC levels of the control group during season 2 (tourist high season for tourist group, Fig 2A). All tests were significant at α = 0.05 with Holm-Bonferroni correction for family wise error in multiple comparisons.

Comparisons between logging and control groups revealed significant main effects of group [*F*(2,129) = 12.0, *p* < 0.001, *r*^2^ = 0.06] and seasonality [*F*(2,129) = 37.4, *p* < 0.0001, *r*^2^ = 0.18].

Investigations on the effect of forest fragmentation with human-wildlife conflicts between chimpanzees from Budongo and Kasongoire forest fragment during comparable seasons revealed significantly elevated HCC in chimpanzees from the fragment group [*F*(1,88) = 5.0, *p* = 0.03, *r*^2^ = 0.20; Fig 2B]. The same result was obtained when only new nests were included into the analysis [*F*(1,33) = 8.1, *p* < 0.01, *r*^2^ = 0.20].

## Discussion

To our knowledge, this is the first study that uses hair cortisol concentrations from shed chimpanzee hairs recovered from their nests to estimate long-term stress levels in response to anthropogenic impacts in wild chimpanzees, considering habituated as well as unhabituated animals. Our results show that HCC can be used to evaluate conservation threats and also permit some methodological conclusions.

### Methodological issues

Due to the fact that this is the first study that uses shed hair from sleeping nests, we need to emphasize the methodological results of this study. HCC stability along the hair shaft revealed waning cortisol concentrations towards the distal end of hair which resembles our results on semi-wild sanctuary chimpanzees [29] and is similar to results on human hair [28]. The present data furthermore resemble our prior results on chimpanzees showing that HCC do not decline towards zero but stabilizes towards an asymptotic concentration that depends on the initial HCC. This indicates that we can limit the influence of this waning effect on our results by using a fixed length of hair within and between samples, which is why we used the proximal 3 cm of hair throughout this study.

We also found a significant effect of nest age on HCC. Nest age and waning effects resemble each other as HCC decrease with time. UV-irradiation and water are most frequently suggested to cause the waning effect [22,29,30,65]. However, all hair samples were collected during dry seasons (even though older nests may have experienced rain) and additionally, shed hairs in nests were only seldom directly exposed to sunlight, especially in older nests where most hair was found beneath the nest surface. Thus, it remains to be investigated whether the HCC decrease in both effects are caused by the same or by different factors. Nevertheless, it is a promising fact that comparing the fragment living chimpanzees with the Budongo chimpanzees we could explain the same amount of variance regardless of whether we used all nests and controlled for nest age or whether we used new nests only (Fig 2). Consequently, we can use hair from older nests, but we have to control for nest age.

A general concern about interpretations on group stress levels from GC hormones arises from our finding that two out of three investigated groups showed significant seasonal differences besides the fact that our hair samples reflected a time interval of approximately three months. Storage time cannot explain this effect because in the Bugoma samples (logging group), earlier samples exhibited higher HCC, compared to the opposite effect in the control group from Budongo forest, and no seasonal differences in the tourist group (Table 1). Nutritional stress could be an explanation, although Newton-Fisher [66] found no seasonal food scarcity in the nearby Sonso community. Despite this finding, forest guides at Kaniyo Pabidi stated that July and August are associated with low fruit availability during which the tourist chimpanzees, that are not food provisioned, move away from their core area into a forest–bush land mosaic where they have access to leguminous fruits. At the same time, it is known from nest sightings that the unhabituated control community partly uses the core area of the tourist community which is then not visited by tourists or guides (Joyse Tuhaise, pers. comm.). While these indications are not enough to prove that the seasonality effect arises from temporal nutritional stress, it highlights the jeopardy to over interpret group differences in GC levels based on only one season, even though hair from chimpanzees represented a three months time period.

One great advantage of monitoring long-term stress through cortisol concentration in shed hair from chimpanzee nests is that it allows investigations on completely non-habituated chimpanzees. However, this advantage comes with a lack in knowledge about the number of individuals that were sampled for most of our groups (except for the fragment community and the tourist community in the second season, Table 1). In fact, it is likely that single individuals were sampled more than once even though we tried to minimize this risk by avoiding the sampling of neighboring nest groups with similar ages. Having no further information about the animals may also be problematic because it does not allow to control for a potential effect of higher HCC in males than in females that was found in semi-wild sanctuary chimpanzees [38], although not for the seven male and seven female chimpanzees of the Kasongoire fragment community in the present study (data not shown). One last inaccuracy of this method derives from the fact that female chimpanzees can share their nests with their infants and juveniles up to an age of seven years [67]. While it seems to be possible to recognize infant hairs that are thinner, shorter and sometimes wavy (own observations) samples from females are likely to be mixed with that of juveniles. However, because females are unlikely to be without a baby or juvenile once they have reached sexual maturity, this inaccuracy should be equal across all groups.

### Conservation results

Our results suggest that chimpanzees which are regularly visited by tourists showed no seasonal differences and were not more stressed than the non-habituated neighboring control community. Instead, the significant interaction effect showed elevated HCC in the control group during the high-tourist season, even though this control group had no contact to tourists at any time. Thus, other group-associated stressors, for example temporary instability of hierarchy, might have a larger impact on chimpanzee group stress levels than tourism in Kaniyo Pabidi. Regarding other great ape studies, Muehlenbein and colleagues [47] showed similar results in two orang-utans, whereas Shutt and colleagues [18] found that western lowland gorillas exhibited slightly increases GC levels in feces due to tourism even if animals were long-term habituated. The same study also revealed that GC levels in the tourism group especially increased if visitors violated the 7 m distance rule. Although systematic observations are missing in our study, our experience was that field guides in Kaniyo Pabidi ensured that visitors would not get closer than 10 m to the chimpanzees as proposed by guidelines for “good ecotourism” [68]. On the other hand, the very conservative restrictions suggested by Williamson and Macfie [68] were not met concerning the maximum number of tourists per group (2 x 6 instead of 4) and number of visits allowed per day (2 or more instead of 1). However, these restrictions are not based on scientific evidence for chimpanzee needs and in addition, chimpanzees may be generally more robust towards the presence of tourists than other great apes, because their fission-fusion-system [69] increases the likelihood that tourists do not always encounter the same animals. Nonetheless, more investigations at different tourism sites and more control groups are needed to draw final conclusions on the effect of ecotourism on chimpanzees.

Our study revealed significantly increased HCC in the Kasongoire fragment community. Activity budgets can be seen as a first indicator on whether or not groups can adjust to adverse living conditions. For example, elevated fecal glucocorticoid levels in black howler monkeys living in a forest fragment was associated with increased travel activity which was interpreted as a reflection of suboptimal food resources [49]. In comparison, Kulp and Heymann [70] found that the activity budget of titi monkeys living in secondary forests and along forest edges were similar. Thus, the authors concluded that forest edges were unproblematic to the animals.

The activity budget of the Kasongoire fragment community (feeding—47%, resting—34%, travelling—11%, grooming each other—3% or themselves—2%, drinking—~1%, playing—~1%, within-group aggression ~0.4%, BCFS unpublished report to WWF, 2012) during the time that is represented by our hair samples was highly comparable with that of other long-studied chimpanzees in intact forests (Gombe: [71]; Taï: [72,73], Sonso-Budongo: BCFS unpublished report to WWF, 2012). Thus, there was no indication of food scarcity or hierarchy instability that could explain the elevated stress levels found in this group. However, unlike chimpanzees in natural environments, the Kasongoire community was regularly observed crop raiding on the sugar cane plantation of Kinyara Sugar Ltd. (21% of their time feeding) as well as frequently raiding private homesteads. While chimpanzees were not charged feeding in the sugar cane plantation, villagers were observed sending their dogs after the chimpanzees, shouting or throwing stones at them. The report furthermore mentions that these chimpanzees have occasionally attacked humans, especially children between 2009 and 2011. Thus, villagers were negatively predisposed towards chimpanzee conservation in this area [BCFS unpublished report to WWF, May 2012]. In sum, the BCFS report strongly support the assumption that the increased stress levels in the Kasongoire community found in the present study may reflect human-chimpanzee conflict, although more data is needed to draw final conclusions. This shows the importance of physiological measures in addition to behavioral data as an indicator of animal well-being.

Regarding the impact of logging activity on Bugoma chimpanzees, we found that these chimpanzees exhibited significantly lower HCC than the control group in Budongo forest. However, we should avoid over-interpreting this result because the actual effect was small and future studies would need to include detailed spatial data on the severity of logging activity in the different sampling areas.

## Conclusion

In conclusion, this study shows that measuring cortisol concentrations from shed hair found in nests is a powerful tool for the assessment of long-term stress levels in wild chimpanzees and can help to understand the severity of anthropogenic impacts regardless of the animal’s habituation status if confounding effects on HCC are considered. Our results suggest that tourism in Kaniyo Pabidi (Uganda) did not lead to significantly elevated stress levels in chimpanzees whereas we found markedly increased HCC, indicating reduced welfare and potentially reduced fitness, in a chimpanzee community that lived in a forest fragment with severe human-wildlife conflicts. We recommend that future investigations always include data collected in multiple seasons and attempt to sample multiple communities in both the treatment and the control categories, so as to account for the currently poorly understood variation in HCC across seasons and groups.

## Supporting Information

S1 Table Raw data on which all statistical analyses in this paper are based on. (TXT)
